# Experimental Wear Behavior Analysis of Coated Spindle Hook Teeth under Real Harvesting Work Conditions

**DOI:** 10.3390/ma14102487

**Published:** 2021-05-11

**Authors:** Yanqing Gu, Hongwen Zhang, Xiuqing Fu, Lei Wang, Zhenyu Shen, Jun Wang, Zhaoyang Song, Longchang Zhang

**Affiliations:** 1 College of Mechanical and Electrical Engineering, Shihezi University, Shihezi 832003, China; guyanqing163@163.com (Y.G.); wl_mac@shzu.edu.cn (L.W.); wj20192009004@163.com (J.W.); zlc20182009101@163.com (L.Z.); 2 Northwest Key Laboratory of Agricultural Equipment, Ministry of Agriculture and Rural Affairs, Shihezi 832003, China; 3 College of Engineering, Nanjing Agricultural University, Nanjing 210031, China; fuxiuqing@njau.edu.cn (X.F.); 2019812052@njau.edu.cn (Z.S.); 2019812053@njau.edu.cn (Z.S.)

**Keywords:** cotton picker, field work, spindle hook teeth, chrome coating, wear

## Abstract

This study aimed to investigate the wear failure changes of spindle hook teeth and the reasons for such failure during field work. Spindle samples were obtained from a fixed position of the spindle bar under different field picking area conditions and combined with the spatial distribution characteristics of cotton bolls in Xinjiang. After cutting a spindle sample, a scanning electron microscope and an energy spectrum analyzer were used to characterize the micromorphology and element composition of the hook tooth surface and cross section under different working area conditions. The wear parameters of the hook teeth were then extracted. The results showed that the thickness of the coating on the surface of the hook tooth used in this study was between 66.1 µm and 74.4 µm. The major chemical element was chromium, with a small amount of nickel. During the field picking process, failure of the coating on the surface of the hook teeth initially appeared on the tooth tip and tooth edge, and then spread to the entire hook tooth surface. The wear failure of the hook teeth resulted from abrasive wear, oxidative wear, and fatigue peeling. As the picking area increased, the wear area of the hook teeth increased exponentially, while the wear width increased linearly. When the field picking area reached 533.33 ha, the maximum change rate of the wear area was 2.33 × 10^3^ µm^2^/ha, and the wear width was 1.84 µm/ha. During field work, the thickness of the coating decreased from the cutting surface to the tooth edge, and the wear rate gradually increased. The wear rate at Position 1 was the slowest, at 0.01 µm/ha, and the wear rate at Position 5 was the fastest, at 0.25 µm/ha.

## 1. Introduction

As an important cash crop, cotton is highly valued by governments worldwide [1,2,3,4]. Xinjiang is the central cotton planting and production area in China. In 2020, the cotton planting area in Xinjiang reached 2501.9 thousand hectares, and the total output was 5.161 million tons [5,6]. A cotton picker, which is a large-scale cotton harvesting machine, is widely used in the cotton harvesting process in Xinjiang due to its high efficiency and low cost. At present, mechanized cotton harvesting is the primary cotton harvesting method in Xinjiang [7,8].

The spindle is the most used core component of a cotton picker, and its performance directly determines the efficiency and quality of cotton pickers in the field [9,10,11]. Considering the complex and harsh environment of field work, hook teeth are prone to wear and failure during field work [12]. To improve the wear resistance of spindles, the method of electroplating chromium coating is generally used at present as a strengthening treatment [13,14,15]. Meng used the spindle material as a substrate, applied chromium and nickel coatings on its surface, and tested the tribological properties of these coatings under different working conditions. Under dry friction conditions, the friction and wear mechanisms of the chromium coating did not conform to the wear behavior of the spindle during field work, while the nickel coating could be used to strengthen the spindle [16,17,18]. Thereafter, researchers observed the wear failure surface morphology of spindle hook teeth via scanning electron microscopy (SEM); they pointed out that the wear failure of spindle hook teeth was caused by abrasive wear [13,19,20,21]. During the observation and element analysis of the cross section of a spindle, researchers found microcracks and hole defects on the coating, and they determined that the major element of the coating is chromium [22]. However, in his research on the wear mechanism of a spindle during field work, Zhang found that the wear failure of spindle hook teeth is primarily caused by the combined actions of abrasive wear and oxidative wear [23]. Zhang adopted random sampling to collect spindle specimens from the field. He disregarded the fact that differences existed in the wear of spindle hook teeth from the same bar at various installation heights during the field picking operation of the cotton picker. He also did not consider the spatial distribution of cotton in Xinjiang. In the current study, spindle samples from field work were obtained from a fixed position on the spindle bar. Then, the hook teeth of the spindle were cut to study changes in the surface and cross section of the hook teeth during field work. No similar report was found in the literature.

The current work combines the spatial distribution characteristics of cotton in Xinjiang with spindle samples obtained under different field picking area conditions from the ninth spindle height position (located at the primary concentrated distribution area of cotton bolls, 519 mm above the ground) on the spindle bar at the front drum of the cotton picker. SEM was used to characterize the microscopic morphology of the surface and the cross section of the hook teeth under different picking area conditions. Energy-dispersive X-ray spectroscopy (EDS) was performed to analyze the elemental composition of the coating, substrate, and worn surfaces of the spindle hook teeth. The wear failure changes of the hook teeth and the reasons for the wear failure of the hook teeth were explored during field work by extracting the wear parameters of the surface and the cross section of the spindle hook teeth under different picking area conditions. This study provides a reference for further exploration of the wear failure mechanism of spindle hook teeth during field work, and provides a basis for strengthening the surface of a spindle hook tooth.

## 2. Work Process of the Spindle in the Field

**Figure 1:**
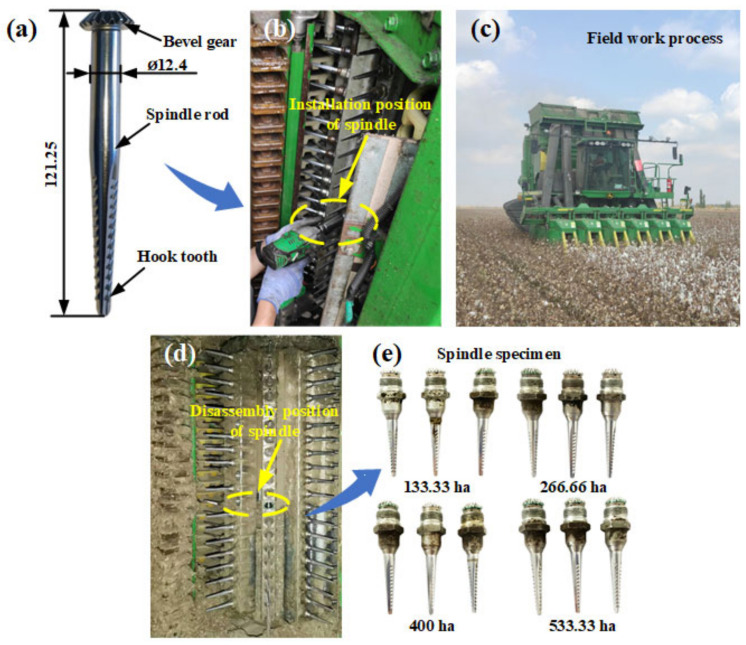
Installation and disassembly process of spindle in the field: (**a**) spindle structure, (**b**) spindle installation process, (**c**) field work process, (**d**) disassembly process of spindle, (**e**) spindle samples.

During the cotton harvesting season in northern Xinjiang in 2020, a certain brand of spindle (the structure of this spindle is shown in Figure 1a), purchased from the market, was installed on a cotton picker (the specific working parameters are provided in Table 1). In accordance with a literature search on the spatial distribution of Xinjiang cotton, Xinjiang cotton bolls are mostly distributed at 250–750 mm, with a concentration of over 95% [24]. In the current study, the installation position of the spindle was selected as the ninth spindle picking position on the spindle bar. During picking, the spindle was 519 mm above the ground and located at the primary height position where the cotton bolls were distributed. Field working day, location, and other information are provided in Table 2.

One of six Pro-16 picking units equipped with a cotton picker was selected as the carrier, a certain brand of spindle was installed at the ninth spindle height position on the front drum bar, and three repetitions were performed for each operation interval. Four different operation intervals were set, and thus, twelve spindles were installed. The installation diagram of the spindles is shown in Figure 1b, and each position is marked. The entire process of tracking and recording the field work operation of the cotton picker is illustrated in Figure 1c. When the picking area of the cotton picker reached 133.33, 266.66, 400, and 533.33 ha, the installed spindles were disassembled. Three spindles were disassembled each time, as shown in Figure 1d. The disassembled spindle samples (Figure 1e) were placed in a sample bag after undergoing anti-rust treatment. The bag was sealed and labeled.

## 3. Materials and Methods

To conveniently study the surface and cross-section wear changes of the spindle hook teeth, we cut the spindle hook teeth and prepared samples. The following sections describe the equipment used and the sample-making process.

### 3.1. Equipment

The following pieces of equipment were used in the study: a cotton picker (equipped with Pro-16 picking units), a scanning electron microscope (model: S-4800, Tokyo, Japan), an energy spectrum analyzer (Bruker AXS, Inc., Berlin, Germany), a wire-cut electric discharge machine (model: DK-7735, Ningbo, China), and an ultrasonic cleaner. The special tools used for the disassembled spindles were as follows: a marker pen, sample bags, absolute ethanol, sandpaper, and a polishing agent.

### 3.2. Cutting Process of Spindles

**Figure 2:**
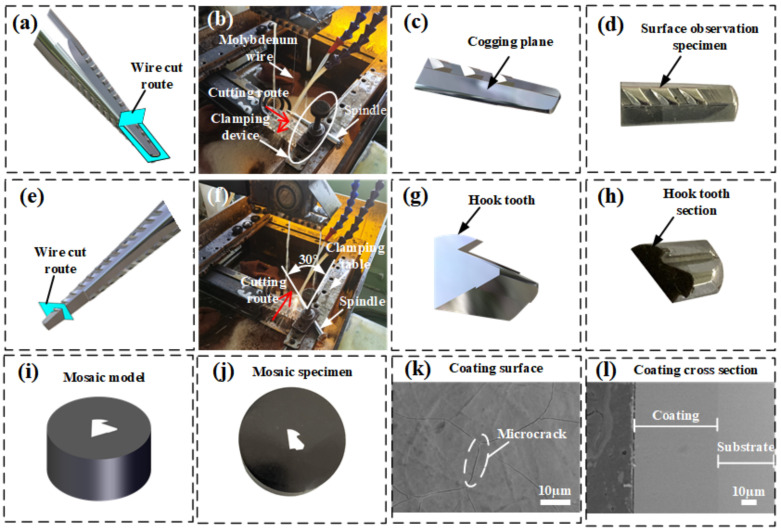
Specimen preparation process: (**a**) schematic of the first wire cutting track, (**b**) first wire cutting process, (**c**) surface observation model of the spindle hook tooth, (**d**) surface observation specimen of the spindle hook tooth, (**e**) schematic of the second wire cutting track, (**f**) second wire cutting process, (**g**) cross-section observation model of the spindle hook tooth, (**h**) cross-section observation specimen of the spindle hook tooth, (**i**) mosaic model, (**j**) mosaic specimen, (**k**) surface coating of the new spindle hook tooth, and (**l**) cross section of the new spindle hook tooth.

The spindle samples obtained from the field were soaked in absolute ethanol for ultrasonic cleaning for 5 min to remove impurities and oil stains on their surface. The samples were then dried with hot air. The first hook tooth of the spindle specimen removed from the field was selected as the research object. The spindle was cut using a wire cutter. The cutting direction is shown in Figure 2a. The spindle cogging plane was adjusted to the vertical direction, and the spindle was clamped. The cutting process is illustrated in Figure 2b, and the wear surface specimen of the spindle hook tooth is shown in Figure 2c,d. After the ultrasonic cleaning and hot-air drying of the wear surface specimen of the spindle hook tooth, an S-4800 scanning electron microscope was used to characterize the wear morphology of the surface of the hook tooth in different work areas, with an accelerating voltage of 15 kV. The first hook tooth of the other row of hook teeth was cut. The cutting direction is shown in Figure 2e. The level of the other cogging plane of the spindle was adjusted. The angle between the axis of the spindle and the clamping table was 30°. The cutting process is illustrated in Figure 2f. The cross section of the specimen obtained from the spindle hook tooth is shown in Figure 2g,h. After mosaicking, grinding, and polishing, the specimens shown in Figure 2i,j were fabricated. After ultrasonic cleaning and hot-air drying, an S-4800 scanning electron microscope was used to characterize the microstructure and thickness changes of the coating cross section of the spindle hook tooth in different working areas. Figure 2k,l show the surface and cross section morphologies of the hook tooth of the new spindle. An EDS energy spectrum analyzer was used to analyze the elemental composition of the coatings, substrates, and worn surfaces; the acceleration voltage was 16 kV, the maximum working distance was 11 mm, and the maximum scanning area was 1 mm^2^.

## 4. Results and Discussion

We cut the spindle to produce specimens that were convenient for microscopic observation and then conducted SEM to characterize the changes in the surface and cross section of the hook teeth during field work. We also analyzed the reasons for the wear failure of the hook teeth. Lastly, we described the wear failure process of the spindle hook teeth by extracting the wear parameters of the hook teeth.

### 4.1. Surface Morphology of the Hook Tooth

**Figure 3:**
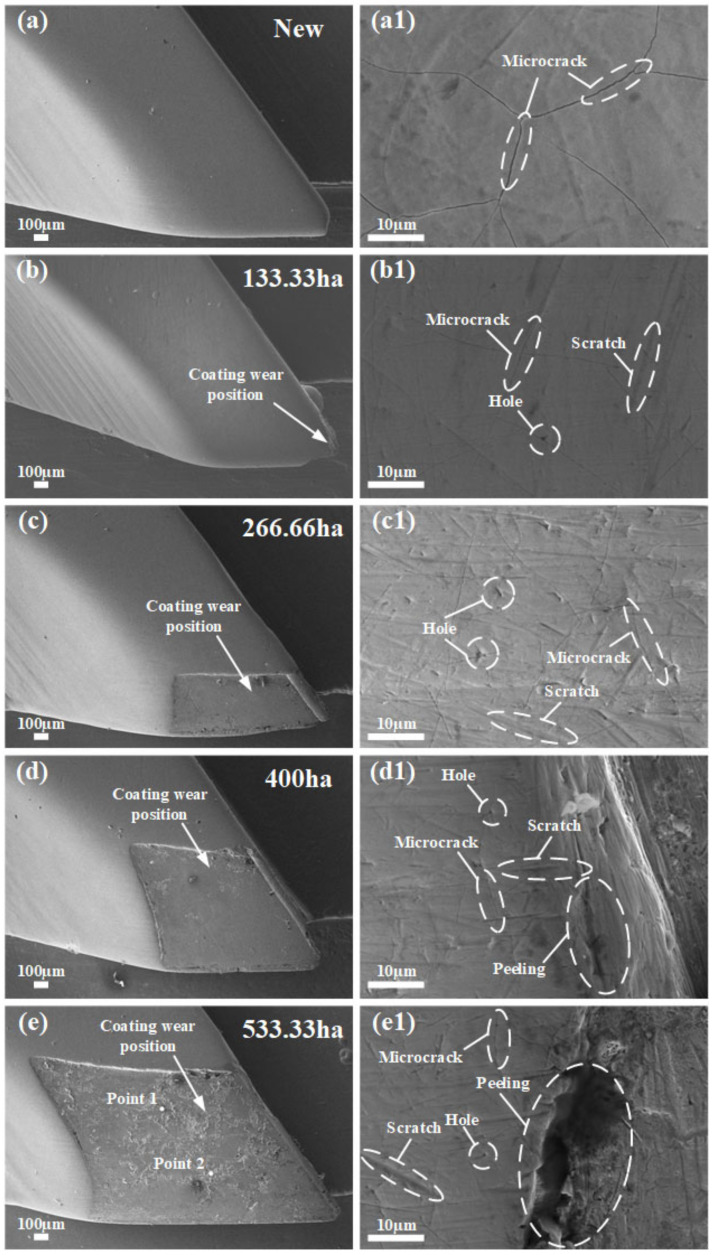

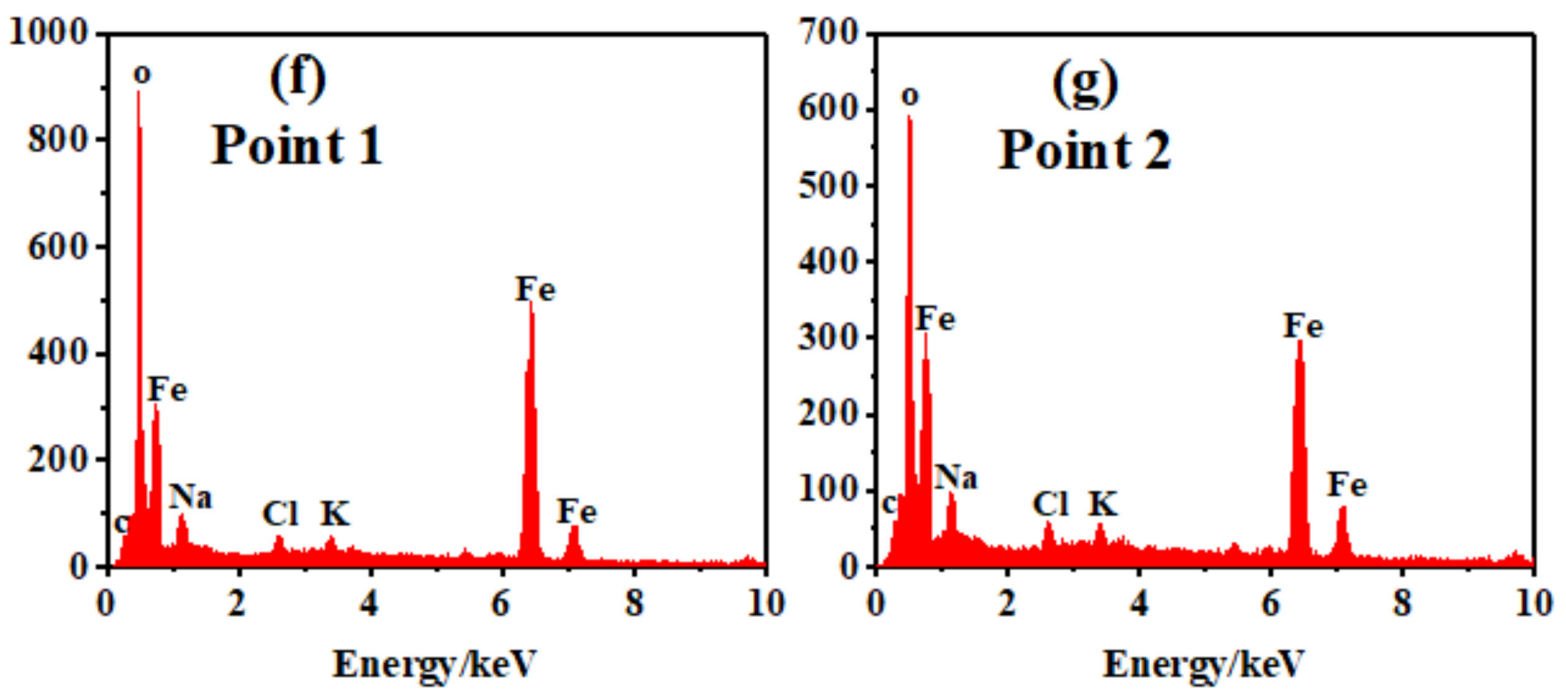
Wear changes of hook teeth during field work: (**a**) hook tooth of the new spindle, (**a1**) surface morphology of the new hook tooth, (**b**) hook tooth of the field picking area with 133.33 ha, (**b1**) surface morphology of the hook tooth of the field picking area with 133.33 ha, (**c**) hook tooth of the field picking area with 266.66 ha, (**c1**) surface morphology of the hook tooth of the field picking area with 266.66 ha, (**d**) hook tooth of the field picking area with 400 ha, (**d1**) surface morphology of the hook tooth of the field picking area with 400 ha, (**e**) hook tooth of the field picking area with 533.33 ha, (**e1**) surface morphology of the hook tooth of the field picking area with 533.33 ha, (**f**) EDS analysis image at Point 1, and (**g**) EDS analysis image at Point 2.

A scanning electron microscope was used to characterize the wear changes and microscopic morphology of the surface of the hook teeth during field work. The results are presented in Figure 3a–e. As shown in Figure 3a, the surface of a hook tooth of the new spindle was completely covered with chromium coating. However, many microcracks occurred on the surface of the coating, as observed in the microscopic morphology of the hook tooth surface shown in Figure 3(a1). The analysis indicated that the existence of these microcracks was related to the inevitable internal stress generated during the electroplating and machining processes [25,26,27,28,29]. After completing 133.33 ha of spindle picking in the field, the coating on the tooth tip and tooth edge exhibited a slight abrasion, as displayed in Figure 3b. Nevertheless, the coating on the tooth edge of the hook tooth was not yet completely destroyed. Noticeable scratches and holes appeared on the surface of the hook tooth’s chrome coating, as shown in Figure 3(b1); this abrasive wear phenomenon was caused by hard particles in the field sliding on the surface of the coating [19,20,21,22,23]. The holes in the chromium coating were related to the precipitation of hydrogen on the cathode during the electroplating process [30,31]. When the picking work area reaches 266.66 ha, the coating on the tooth edge of the picking hook tooth was completely destroyed. Meanwhile, the coating on the surface of the spindle hook tooth presented a trapezoidal wear area that exposed the substrate material, as shown in Figure 3c. The number of scratches on the surface of the hook tooth’s chrome coating increased and became more complex, as shown in Figure 3(c1). When the field picking work area reached 400 ha, the trapezoidal wear area on the surface of the spindle hook tooth was evidently enlarged, as illustrated in Figure 3d. At this moment, the phenomenon of peeling of the coating on the surface of the hook tooth was observed, as shown in Figure 3(d1). From the analysis, the spindle was assumed to collide with harder materials, such as cotton stalks and bell shells, during the field picking process. Through the continuous collision with such hard materials, the fatigue life of the chrome coating was reduced, and fatigue peeling was likely to occur [21,32,33,34,35,36,37], accelerating the wear rate of the coating on the surface of the spindle. When the picking work area reached 533.33 ha, the trapezoidal wear area on the surface coating of the hook teeth increased significantly, as shown in Figure 3e. The fatigue peeling phenomenon of the coating was further aggravated, as depicted in Figure 3(e1). The hardness of the spindle substrate was considerably lower than that of the coating, and thus, the hook tooth substrate without the protection of the coating underwent rapid wear and lost its original structure, eventually leading to spindle failure [12].

An energy spectrometer is an important auxiliary instrument of a scanning electron microscope. It can complete the qualitative and quantitative analyses of the elements in the microscopic area of a material within a short period. In the current study, an X-ray spectrometer was used to study the distribution of elements on the wear surface of the spindle hook tooth. Point scanning was performed on Points 1 and 2 of the worn surface of the spindle. The scanning position is shown in Figure 3e. The scanning results are presented in Figure 3f,g. The element composition at Points 1 and 2 is mostly oxygen and iron, with a small amount of sodium, potassium, and chlorine, indicating that the substance at Points 1 and 2 is an iron oxidation product [23,38,39,40]. After the coating on the surface of the hook tooth wears and fails, the substrate is exposed to air, and the surface of the substrate exhibits oxidized wear, accelerating the wear failure of the hook tooth substrate [41,42,43].

**Figure 4:**
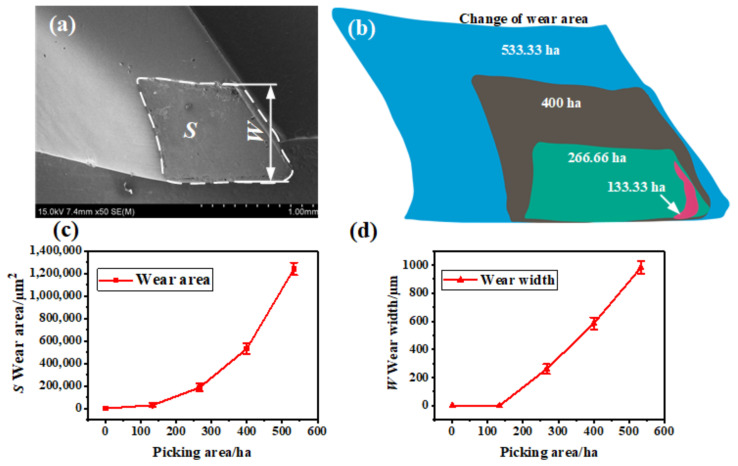
Wear change on the surface of the spindle: (**a**) schematic of wear area and wear width, (**b**) wear area extraction result, (**c**) change curve of wear area, and (**d**) change curve of wear width.

To provide a clear description of the wear change process of the hook tooth of the spindle, the wear width and wear area of the hook teeth were used as indicators to describe the wear degree of the hook teeth. The wear area refers to the wear area of the hook tooth section of the spindle, as shown in Figure 4a. It is represented by *S* The wear width refers to the closest distance between the worn part of the hook tooth edge and the unworn part of the coating, as depicted in Figure 4a. It is represented by *W* The wear area and wear width of the spindle hook teeth were extracted using an image processing method. Figure 4b shows the changes in the wear area of the spindle hook teeth under different field work area conditions. The wear area of the spindle hook teeth continued to increase with the increase in field work area. The change curve of the wear area and wear width is presented in Figure 4c,d. The wear area and wear width of the hook tooth of the new spindle were both 0. When the field picking area reached 133.33 ha, the wear area was 2.86 × 10^4^ µm^2^. At this moment, no wear width matched the definition in the text, and the wear width was still 0 µm, because the wear on the surface coating of the hook tooth occurred on the tooth tip and tooth edge. At this moment, the wear area change rate was 214.51 µm^2^/ha. When the field picking area reached 266.66 ha, the wear area increased to 1.86 × 10^5^ µm^2^, and the wear width was 259.92 µm. The wear area and wear width change rate were 697.52 µm^2^/ha and 0.97 µm/ha, respectively. When the field picking area reached 400 ha, the wear area was 5.32 × 10^5^ µm^2^, and the wear width was 583.83 µm. The wear area and wear width increased significantly, and the change rates of the wear area and wear width were 1.33 × 10^3^ µm^2^/ha and 1.46 µm/ha, respectively. When the field picking area reached 533.33 ha, the wear area was 1.24 × 10^6^ µm^2^, and the wear width was 981.49 µm. The change rate of the wear area was 2.33 × 10^3^ µm^2^/ha, and that of the wear width was 1.84 µm/ha. The preceding data indicate that the wear failure rate of the surface of the hook tooth of the spindle was accelerated with an increase in the field picking area. The reason for this finding was as follows. On the one hand, the fatigue peeling phenomenon of the coating on the surface of the hook tooth of the spindle is increased with an increase in the field picking area, and the wear failure rate of the coating is accelerated [21,32,33,34,35,36,37]. On the other hand, the substrate is exposed after the coating is worn out, and the appearance of oxidative wear on the surface of the substrate further accelerates the wear rate of the hook teeth [41,42,43]. As shown in Figure 4c,d, the wear area of the spindle hook tooth during the picking process exhibited an exponential trend. The wear width of the spindle hook tooth presented an approximately linear relationship with the area of field picking operation.

### 4.2. Cross Section Morphology of the Hook Tooth

**Figure 5:**
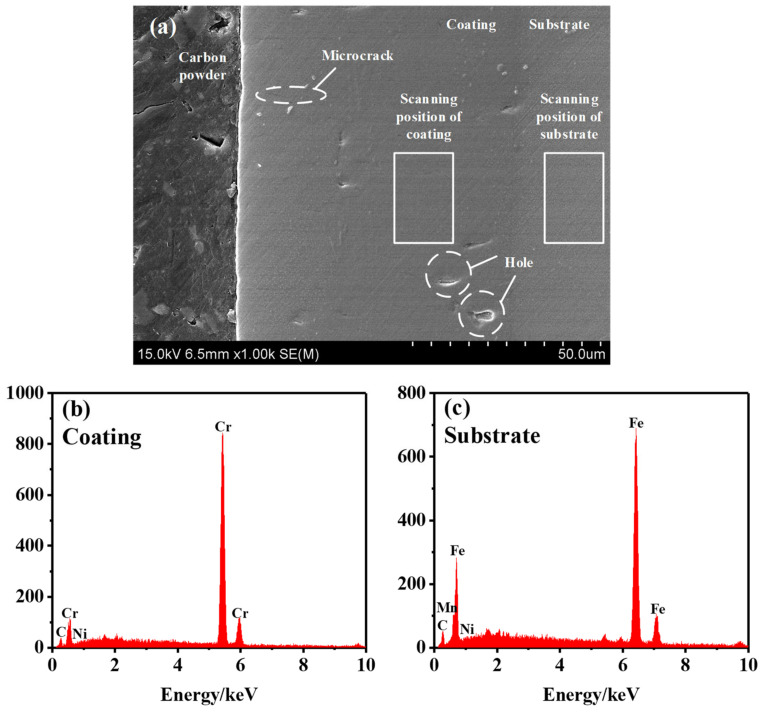
EDS analysis of the coating and substrate: (**a**) cross-section morphology of the hook tooth of the spindle, (**b**) coating analysis results, and (**c**) substrate analysis result.

The spindle hook teeth were cut, and SEM was performed to characterize the cross-section morphology of the coating of the spindle hook teeth. The results are presented in Figure 5a. As shown in the figure, microcracks and hole defects occurred in the chromium coating of the new spindle [22]. Through the analysis, the existence of microcracks in the chromium coating was assumed to be related to inevitable internal stress during the machining and electroplating processes [25,26,27,28,29]. The holes were caused by the precipitation of hydrogen at the cathode during the electroplating process, and hydrogen bubbles were formed on the surface of the coating [30,31].

EDS was performed to scan the coating and substrate of the hook tooth, with a scanning area of 0.01 mm^2^, as shown in Figure 5a. Figure 5b,c present the EDS spectra of the coating and substrate of the hook tooth. Chromium and nickel were detected in the coating; while iron, manganese, and nickel were present in the substrate [12]. The content of each element in the coating and substrate is provided in Table 3. The major element in the coating was chromium [22], with a content of 95.60%. The content of nickel was less, i.e., 4.40%. The primary element in the substrate was iron, with a content of 91.48%. Meanwhile, the contents of manganese and nickel were less, namely, 6.08% and 2.44%, respectively.

**Figure 6:**
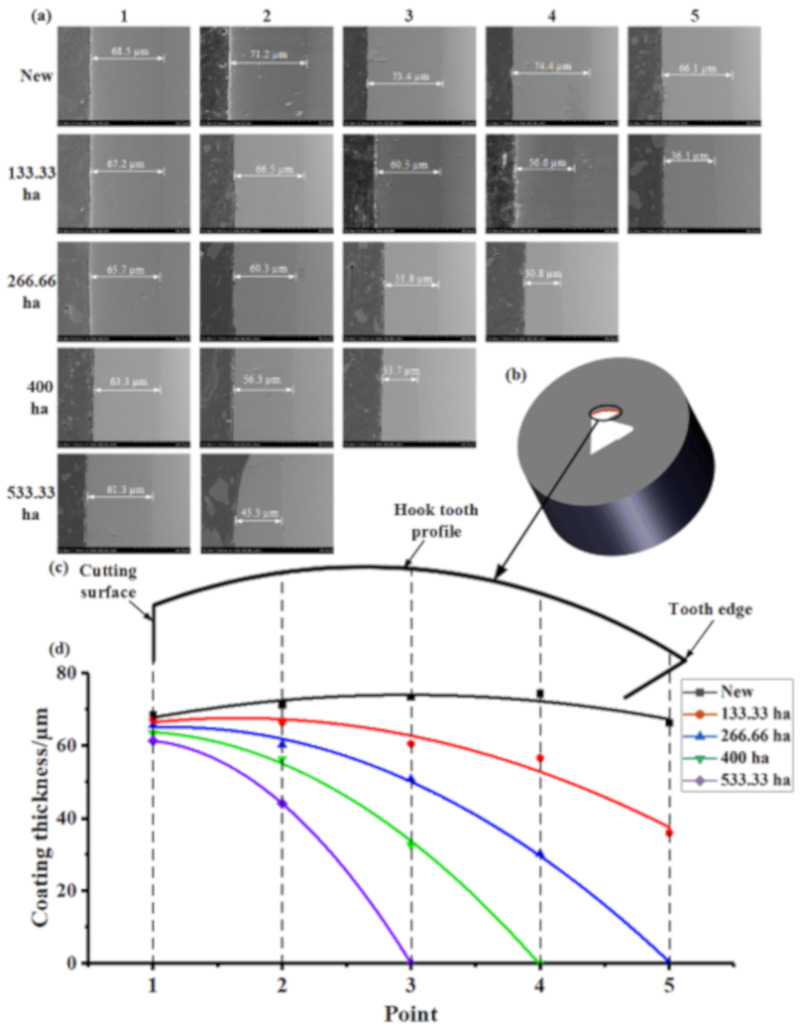
Change in coating thickness of the hook tooth surface during field picking: (**a**) thickness of the coating on the surface of the spindle at each position, (**b**) coating thickness measurement specimen, (**c**) hook tooth profile of the spindle, and (**d**) change curve of coating thickness on the surface of the hook tooth of the spindle.

The cutting surface was set as Position 1. The remaining four points were determined at intervals of approximately 0.3 mm, as shown in Figure 6b,c. The thickness of the coating on the surface of the hook tooth of the spindle was measured at each position, as illustrated in Figure 6a. Figure 6c presents the change in the thickness of the coating on the surface of the spindle hook teeth during field picking. The surface coating of the hook teeth of the new spindle was thick in the middle but thin on both sides. The thickness of the coatings at Positions 1–5 of the new teeth was 68.5, 71.2, 73.4, 74.4, and 66.1 µm, respectively. Under different picking area conditions, the thickness of the coating on the surface of the same hook tooth gradually decreased from Positions 1 to 5. The curve of the coating thickness changed more evidently as the area of field picking operations increased. In the same position, the thickness of the coating continued to decrease as the area of field picking operations increased [23]. The wear failure rate of the coating near the cutting surface was slower, and the wear rate near the tooth edge was faster, as shown in Figure 6d. The wear rates of the coating at Positions 1 (cutting surface), 2, 3, 4, and 5 (tooth edge) were 0.01, 0.05, 0.14, 0.19, and 0.25 µm/ha, respectively. The wear rate gradually decreased from Positions 1 to 5. The wear rate of Position 1 was the slowest, and that of Position 5 was the fastest.

## 5. Conclusions

In the current study, a certain brand of spindle purchased from the market was installed at a fixed position in a cotton picker and spindle samples were obtained from different field work areas. The wear change of the surface and cross section of the hook teeth of the spindle were studied, and the reasons for the wear failure of the hook teeth were analyzed. The following conclusions were drawn.

1. The analysis of the surface of the spindle hook tooth showed that during field work, the wear on the surface of the spindle hook tooth initially occurs at the tooth tip and tooth edge, and then gradually spreads to the entire hook tooth surface. The wear area of the hook teeth increases exponentially with an increase in field work area, and the wear width changes linearly. In this study, when the working area of the field work section reached 533.33 ha, the maximum wear area and wear width change rates were 2.33 × 10^3^ µm^2^/ha and 1.84 µm/ha, respectively.
2. Through the analysis of the wear failure of the spindle hook teeth, we determined that the wear of the spindle hook teeth was caused by the combined actions of abrasive wear, oxidative wear, and the fatigue peeling of the coating.
3. In the analysis of the cross section of the spindle hook teeth, we found microcracks and holes in the spindle coating used in this study. The thickness of the coating on the tooth edge was small, and the thickness of the surface coating of the hook tooth was between 66.1 µm and 74.4 µm.
4. During field work, the thickness of the coating on the surface of the same spindle hook tooth gradually decreased from the cutting surface to the tooth edge at different positions. However, the surface coating of the hook tooth at the same position exhibited a slower wear rate near the cutting surface under the conditions of different work areas, whereas the wear rate of the coating near the tooth edge was faster. In this study, the wear rate at Position 1 was the slowest, at 0.01 µm/ha, and the wear rate at Position 5 was the fastest, at 0.25 µm/ha. The wear failure change of the hook teeth and the reasons for the wear failure of the hook teeth were explored during field work, and a reference for further exploration of the wear failure mechanism of spindle hook teeth is provided. However, this study only analyzed the wear of the first hook tooth of the spindle. The wear of the remaining hook teeth of the spindle will be studied in follow-up research.

## Figures and Tables

**Table 1:** Working parameters of the cotton picker.

Parameter	Field Speed (km/h)	Front Drum Speed (rpm)	Spindle Speed (rpm)	Doffer Pads Speed (rpm)
Value	0–7.1	0–152	0–4652	0–1960

**Table 2:** Working conditions of the field.

Picking Location	Picking Date	Picking Time	Temperature (°C)	Humidity (%)
Machine-picked cotton in Kuitun, Xinjiang, China	2020.9.25–2020.10.18	8:00 a.m.–12:00 p.m.	−2.00–18.50	22.62–67.92

**Table 3:** Element content in the coating and substrate.

Element	Fe Content (wt%)	Cr Content (wt%)	Mn Content (wt%)	Ni Content (wt%)
Coating	―	95.60	―	4.40
Substrate	91.48	―	6.08	2.44

## Data Availability

All relevant data presented in the article are stored according to institutional requirements and, as such, are not available online. However, all data used in this manuscript can be made available upon request to the authors.
